# HIV-1 Nef inhibits the JAK/STAT signaling pathway by inducing proteasomal degradation of STAT1

**DOI:** 10.1371/journal.pone.0352649

**Published:** 2026-07-10

**Authors:** Roger L. Rodrigues, Mara E. da Silva-Januário, Vinicius B. Apolloni, Constanza E. Espada, Taissa R. Jorge, Lucas A. Tavares, Andreia N. de Carvalho, Juliano P. Souza, Eurico Arruda, Iranaia Assunção-Miranda, Daniel S. Mansur, Luis L. P. daSilva

**Affiliations:** 1 Center for Virology Research and Department of Cell and Molecular Biology, Ribeirão Preto Medical School, University of São Paulo, Ribeirão Preto, São Paulo, Brazil; 2 Laboratory of Immunobiology, Department of Microbiology, Immunology and Parasitology, Center of Biological Sciences, Federal University of Santa Catarina, Santa Catarina, Brazil; 3 Department of Virology, Paulo de Góes Institute of Microbiology, Federal University of Rio de Janeiro, Rio de Janeiro, Brazil; University Hospital Tuebingen, GERMANY

## Abstract

Type I interferon (IFN-I) is critical for controlling viral infections through the activation of the JAK/STAT signaling pathway, which drives the transcription of interferon-stimulated genes (ISGs) with diverse antiviral functions. Despite its importance, the effectiveness of IFN-I treatment against HIV-1 is limited. The HIV-1 accessory protein Nef is highly expressed early during infection and is detectable in the serum of HIV-1-infected individuals. Furthermore, Nef is a key factor in viral pathogenesis and disease progression, that has been shown to antagonize specific ISG products via post-translational mechanisms. To investigate if Nef plays a broader effect in antiviral responses, we used a T cell line constitutively expressing Nef and analyzed the JAK/STAT pathway activation after IFN stimuli. Here, we demonstrate that Nef interferes with the JAK/STAT signaling pathway by selectively depleting STAT1 in T cells. This Nef-mediated depletion depends on Nef myristylation and proteasomal activity but not on lysosomal activity. In contrast, the levels of STAT2, an interaction partner of STAT1, as well as the phosphorylation of upstream kinases JAK1 and TYK2, remain unaffected by Nef. Importantly, the depletion of STAT1 in Nef-expressing T cells compromises the induction of antiviral ISGs by IFN-α. These findings reveal a novel role for Nef in T cells, suggesting a mechanism through which HIV-1 evades IFN-α-induced antiviral responses, potentially contributing to viral immune evasion.

## Introduction

HIV-1/AIDS remains a significant global public health concern, affecting millions of individuals worldwide, despite substantial advancements in antiretroviral therapy [1]. The primary targets of HIV-1 infection are T lymphocytes [2,3] and monocyte/macrophages [4], which are essential cells for an effective immune response to control viral infection [5]. During the HIV-1 replication cycle, Pattern-Recognition Receptors (PRR) identify viral components and induce the production and secretion of type I and type III interferons (IFN-I and IFN-III), key mediators of antiviral immunity [6–10]. Notably, during the acute phase of HIV-1 infection individuals exhibit significantly elevated serum levels of type I interferon (IFN-α/β) [11], which likely originates mainly from plasmacytoid dendritic cells (pDCs) [12–14].

IFN-α initiates the JAK/STAT signaling pathway through its dedicated surface receptors, IFNAR1 and IFNAR2 [15]. This process triggers a series of signaling events that include the phosphorylation of receptor kinases, namely Janus-associated kinases 1 (JAK1) and Tyrosine kinase 2 (TYK2), and subsequent phosphorylation of the Signal Transducer and Activator of Transcription 1 and 2 (STAT1/2) proteins [16,17]. This pathway induces the expression of hundreds of interferon-stimulated genes (ISG), promoting an antiviral state in infected and neighboring cells [17,18]. While ISGs are critical for rendering cells refractory to viral infection, viruses have evolved various mechanisms to evade or subvert the host IFN-I response [19]. For HIV-1, the accessory proteins Nef and Vif were previously implicated in JAK/STAT signaling pathway downmodulation [20,21].

Nef is an N-myristoylated accessory protein (27−35 kDa) of HIV-1, HIV-2, and SIV that lacks enzymatic activity. This multifunctional protein facilitates HIV-1 immune evasion and contributes to pathogenesis by modifying the intracellular environment of infected cells to support viral replication [22]. Additionally, Nef is present either freely or in extracellular vesicles (EVs) in the serum of infected humans and non-human primates, even during suppressive antiretroviral therapy (ART) [23–27]. Importantly, exogenous Nef internalized by recipient cells remains biologically active and has been shown to induce pro-apoptotic signaling cascades in CD4^+^T cells [28], disrupt cholesterol efflux through downregulation of ABCA1 [29], alter lipid raft organization, which potentiate the release of pro-inflammatory cytokines such as TNF-α [30] and to induce myelin disruption and oligodendrocyte damage in the central nervous system of mice [31].

Nef is widely recognized for its ability to modulate several signaling cascades, both when expressed independently and during infection. Notably, Nef interacts with proteins involved in kinase signaling pathways, influencing signaling cascades activated by non-receptor tyrosine kinases, including those in the Src and Tec families (reviewed in [32]). Nef’s modulation of these pathways is facilitated by interactions between Nef homodimers and plasma membrane–associated tyrosine kinases, primarily through a conserved proline-rich motif (PxxPxR) that engages SH3 domains of Src family kinases (e.g., Hck and Lyn) and several Tec family members (e.g., Itk and Bmx) [33–36], notably, the interaction with Bruton’s tyrosine kinase (Btk) occurs independently of this proline-rich motif [37]. These interactions result in enhanced phosphorylation of the targeted kinases, which disrupts host cell signaling networks, contributing to viral replication and pathogenesis [32]. Additionally, the JAK/STAT signaling pathway, activated by the JAK family of tyrosine kinases (JAK1, JAK2, JAK3, and TYK2), plays a critical role in eliciting a robust anti-viral immune response in both infected and uninfected cells (reviewed in [38]). Previous studies have suggested that Nef also modulates JAK/STAT pathway activation by reducing phosphorylated STAT1 levels in interferon-stimulated CD4^+^ T lymphocytes [20]. However, the precise mechanism underlying this activity remains to be elucidated.

In this study, we investigated how Nef counteracts the JAK/STAT pathway. We demonstrate that Nef promotes proteasome-mediated degradation of STAT1, resulting in a marked reduction in total STAT1 levels in T cells. Consequently, IFN-α stimulation fails to induce the normal accumulation of phosphorylated STAT1, likely impairing the formation of the IFN-stimulated gene factor 3 (ISGF3) complex, a transcription factor composed of STAT1, STAT2, and IRF9. Notably, Nef does not impair phosphorylation of upstream kinases JAK1 and TYK2 nor does it alter total STAT2 levels, indicating that it selectively targets STAT1. Furthermore, STAT1 depletion requires Nef membrane association and is conserved among Nef alleles from the laboratory HIV-1 strain NL4−3, the primary HIV-1 isolate NA7, and SIVmac239, suggesting that this mechanism is evolutionarily conserved among primate lentiviruses. By depleting STAT1, Nef disrupts the JAK/STAT pathway, thereby suppressing IFN-α-mediated signaling and the induction of antiviral ISG in CD4^+^ T lymphocytes.

## Materials and methods

### Cell culture

A3.01 CD4^+^ T cells, obtained from the National Institutes of Health (NIH) AIDS Research and Reference Reagent Program (Germantown, MD, USA) and originally deposited by Thomas Folks, were cultured in RPMI 1640 medium (Life Technologies, Carlsbad, CA, USA) supplemented with 100 U/mL penicillin, 0.1 μg/mL streptomycin, 2 mM L-glutamine, and 10% fetal bovine serum (FBS) at 37 °C and 5% CO_2_. PEAK cells (HEK-293-P), kindly provided by Dr. Reuben Siraganian (NIH, Bethesda, MD, USA), were maintained in Dulbecco’s modified Eagle medium (DMEM) (Life Technologies, Carlsbad, CA, USA) supplemented with 100 U/mL penicillin, 0.1 μg/mL streptomycin, 2 mM L-glutamine, and 10% FBS at 37 °C and 5% CO_2_.

### Expression Vectors and Reagents

The pMSCV-internal ribosome entry site (IRES)-GFP and pMSCV-Nef NL4−3 WT-IRES-GFP retroviral vectors, expressing a Nef from NL4−3 strain, were previously described [39]. A pMSCV-IRES-GFP-based vector expressing the Nef NL4−3 G2A mutant, where a Glycine amino acid residue is replaced by Alanine residue at position 2 of Nef NL4−3, was generated. To this end, the HIV-1 Nef G2A or the Nef SIVmac 239 ORFs in the pIRES2-eGFP plasmid [40] were amplified by PCR flaked by *BglII* and *SalI* restriction sites. These DNA fragments were then transferred into the plasmid pMSCV-IRES-GFP, using restriction enzymes *BglII* and *SalI*, obtaining pMSCV-NefG2A-IRES-GFP and pMSCV-NefSIVmac239-IRES-GFP plasmids. The pMSCV-NefNA7-IRES-GFP plasmid to express the primary Nef allele NA7, isolated from a human HIV-1 infected individual [41], was previously described [42]. All constructs were confirmed by sequencing.

### Transduction of A3.01 T Cells for expression of GFP, NefWT/GFP, NefG2A/GFP or NefNA7/GFP

Retroviruses coding Nef alleles and GFP, or GFP alone, were produced using the retroviral system plasmids pCL-Eco and pVSV-G. For this, PEAK cells (4 × 10^6^) in a 100 mm cell culture dish were co-transfected with 3 μg of pVSV-G, 6 μg of pCL-Eco, and 9 μg of either pMSCV-IRES-GFP, pMSCV-NefWT-IRES-GFP, pMSCV-NefG2A-IRES-GFP, pMSCV-NefNA7-IRES-GFP or pMSCV-NefSIVmac239-IRES-GFP plasmids, using 30 μL of 25 kDa linear polyethyleneimine (PEI) (1 µg/µl stock solution) transfection reagent (Polysciences Inc, Warrington, PA, USA). The cell supernatant containing retrovirus was collected 36 h after transfection, clarified by centrifugation (2,000 × *g* 10 minutes, 4 °C) and used to infect A3.01 CD4^+^ T lymphocytes for 24 h. Transduced cells were washed with PBS and cultured for 72 h in complete RPMI media. GFP-positive cells were sorted using FACSAria III (BD, Franklin Lakes, NJ, USA). After sorting, cells were expanded, and homogeneous GFP and Nef expression was confirmed by flow cytometry and western blot analysis before being used in experiments. The resulting cell populations were consistently >95% GFP-positive.

### Cell Viability and Flow Cytometry Analysis

BD Horizon Fixable Viability Stain 575V (FVS575V) was used to determine Cell viability. The FVS575V working solution was prepared by diluting 1 μL of FVS575V stock solution [200 μg of FVS575V in 340 μL of fresh cell culture-grade dimethyl sulfoxide (DMSO) (Sigma-Aldrich, San Luis, MI, USA)] in 1 mL of PBS. A3.01 CD4^+^ T lymphocytes expressing GFP, NefWT/GFP, NefG2A/GFP and NefNA7/GFP (1 × 10^6^ cells) were cultured in RPMI supplemented with 10% FBS. Cells were then washed twice with ice-cold PBS, centrifuged at 300 × *g* for 5 minutes at 4 °C, and incubated with 100 µL of ice-cold FVS575V working solution for 15 minutes at 4 °C. Subsequently, the cells were analyzed on the FACSymphony™ A1 Cell Analyzer (BD, Franklin Lakes, NJ, USA) at the Regional Blood Center of Ribeirão Preto – Center for Cell-based Therapy. As gate strategy, we first selected cells using side scatter and forward scatter parameters, then selected singlets and live cells (negative for FVS575V). The FloJo (https://www.flowjo.com/) software was used for data analyses.

### Treatment of CD4^+^ A3.01 T lymphocytes with drugs

For time-course of IFN-I stimuli experiments, CD4^+^ A3.01 T lymphocytes expressing GFP alone or with different Nef alleles (1 × 10^6^ cells/mL) were cultured in RPMI-10% FBS at 37 °C with IFN-α2b (1,000 IU/mL; Bioalbra, 11343516, Viçosa, Brazil) for 5, 10, or 30 minutes (short-term stimuli) or 2, 8, or 24 h (long-term stimuli), as indicated in the Figures. After incubation, cells were harvested and processed for SDS-PAGE and western blot. The drug regimens used to inhibit lysosomal, proteasomal, or immunoproteasomal activity are shown in S3 Fig. CD4^+^ T lymphocytes were incubated for a total of 24 h in RPMI medium with 1,000 IU/mL of IFN-α2b. For lysosomal inhibition, cells were first incubated in RPMI for either 24 h or 16 h with or without IFN-α2b, followed by 8 h in RPMI supplemented with bafilomycin A1 (100 μM; Sigma-Aldrich, B1793) or bafilomycin A1 and IFN-α2b (S3A Fig). To inhibit proteasomal activity, cells were incubated in RPMI for either 24 h or 18 hwith IFN-α2b, then for 6 h in RPMI with MG-132 (20 μM; Sigma-Aldrich, C2211) or with MG132 and IFN-α2b, replacing the medium after 3 h (S3B Fig). For immunoproteasomal inhibition, A3.01 Nef WT/GFP cells were incubated in RPMI without or with IFN-α2b for the whole period of 24 h or for 18 h or 22 h and treated with OXN-0914 (200 nM; ApexBio) and IFN-α2b for 6 or 2 h, respectively (S3C-D Figs).

### SDS-PAGE and immunoblot analysis

Cells were treated as indicated, washed two times with ice-cold PBS, and then lysed with the cell lysis buffer (1mM EDTA, 1M Tris-HCL (pH7.5), and 1% (v/v) Triton X-100) supplemented with a protease inhibitor cocktail (Sigma-Aldrich, San Luis, MI, USA). For SDS-PAGE and immunoblot analysis of phospho-proteins, the lysis buffer (1mM EDTA, 1M Tris-HCL (pH7.5), 1% (v/v) Triton X-100) is supplemented with 100 mM Sodium Orthovanadate, 500 mM Sodium Fluoride, 100 mM Sodium pyrophosphate, 0.27 M Sucrose and a protease inhibitor cocktail (Sigma-Aldrich, San Luis, MI, USA). The cell lysate was centrifuged at 13,000 × *g* for 20 minutes, the supernatant was recovered, and samples were equalized using the Bio-Rad Protein Assay Dye Reagent Concentrate (Bio-Rad, Hercules, CA, USA), mixed with 2x sample buffer [4% SDS, 160 mM Tris-HCl (pH 6.8), 20% glycerol, 4% β-Mercaptoethanol and 0.005% Bromphenol Blue] and heated (95 ºC, 5 minutes). Proteins were resolved by SDS-PAGE under denaturing conditions and transferred to a nitrocellulose membrane (Millipore, Bedford, MA). Membranes were blocked with PBS-T (PBS, 0.5% Tween 20) and 5% nonfat dry milk, followed by incubation with primary antibodies overnight at 4 °C and secondary antibodies for 1 h at room temperature, and detected using a solution of enhanced chemiluminescence (ECL) and visualized with ChemiDoc imaging systems (GE Life Science, Chicago, IL, USA).

### Immunofluorescence and confocal microscopy

CD4^+^ A3.01 T lymphocytes expressing GFP alone or NefWT/GFP (2 × 10^5^ cells/mL) were adhered to 13-mm-diameter glass coverslips using Poly-Lysine solution (Sigma-Aldrich, San Luis, MI, USA). After adhesion, cells were treated with 1,000 IU/mL of IFN-α2b (Bioalbra, Viçosa, MG, Brazil) for 30 minutes or left untreated to induce STAT1 phosphorylation. After treatment, cells were fixed with ice-cold absolute methanol for 3 minutes and blocked with pork gelatin (0.2% [wt/vol]) (Sigma-Aldrich, San Luis, MI, USA) at room temperature. Cells were then incubated with primary and secondary antibodies conjugated to Alexa fluorophores in a blocking solution (0.2% [w/v] pork skin gelatin in PBS) at 37 °C for 30 minutes. After incubation, coverslips were mounted on slides using Fluoromount G (Electron Microscopy Sciences, Hatfield, PA, USA). Cells imagens were captured on an Olympus BX40 (Tokyo, Japan) and post-acquisition image processing was performed using ImageJ (https://fiji.sc/).

### Antibodies

For immunofluorescence labeling, the mouse monoclonal primary antibody STAT1 (phosphor Y701) [M135] (Abcam - ab29045, 1:100) was used together with the secondary antibody conjugated to Alexa 594 fluorophore (Thermo Fisher Scientific - A21203, 1:1,000). For immunoblotting, the following commercially obtained antibodies were used: STAT1 (Cell Signaling Technology, 9172S, 1:1,000), STAT1 (phospho Y701) [M135] (Abcam - ab29045, 1:1,000), STAT2 (Cell Signaling Technology - 72604S, 1:1,000), pJAK1 (Tyr1022, Tyr1023) (Thermo Fisher Scientific – 44-422G, 1:250), pTYK2 (Tyr1054/1055) (Cell Signaling Technology - 9321S, 1:250), USP18 (Cell Signaling – 4813, 1:1,000); ISG15 (R&D System, MAB4845, 1:1,000), IFIT3 (Proteintech – 15201–1-AP, 1:1,000), Ubiquitin (Cell Signaling Technology - sc8017, 1:1,000), CD4 (H-370) (Santa Cruz Biotechnology – sc-72191, 1:1,000), β-actin (Thermo Fisher Scientific - MA1–91 399, 1:1,000) and GAPDH (Sigma-Aldrich - G9545, 1:3000). The anti-Nef rabbit polyclonal antiserum was obtained from NIH AIDS Research and Reference Reagent Program (originally deposited by Ronald Swanstrom) and used as a 1:2000 dilution. The anti-GFP rabbit polyclonal antiserum was kindly provided by R. Hedge (MRC, Cambridge, England, 1:1,000 dilution). The secondary antibodies Horseradish-peroxidase-conjugated donkey anti-rabbit IgG (NA934V) and donkey anti-mouse immunoglobulin G (IgG) were obtained from GE Healthcare.

### Quantification of mRNA expression

CD4^+^ A3.01 T lymphocytes expressing GFP alone or NefWT/GFP (1 x 10^6^ cells/mL) were treated with 1,000 IU/mL of IFN-α2b for 8 h or left untreated, as a control. After treatment, total RNA was extracted using the TRIzol™ Reagent protocol (Thermo Fisher Scientific, Waltham, MA, USA), and cDNA was generated using 1 μg of RNA with the High-Capacity cDNA Reverse Transcription Kits (Applied Biosystems, Foster City, CA, USA). Real- time PCR was performed with equal amounts of cDNA using the SYBER amplification protocol with the qPCRBIO SyGreen Mix Separete-ROX kit (PCR BIOSYSTEM, Wayne, PA, USA), according to the manufacturer’s instructions. GAPDH was used to control housekeeping genes. *IFIT1*, *ISG15*, and *STAT1* mRNA were calculated using the 2^ΔΔ^CT method.

### Statistical analysis

All data were analyzed using GraphPad Prism 8.0 version software (GraphPad Software, La Jolla, CA, USA) and are demonstrated as mean ± SD from at least three independent experiments, as stated in the Figure legends. Band densitometry values were calculated in relation to the GAPDH housekeeping protein signal using imageLab software 6.1.0 version (Bio-Rad Laboratories).

## Results

### HIV-1 Nef protein inhibits ISG expression

**Fig 1:**
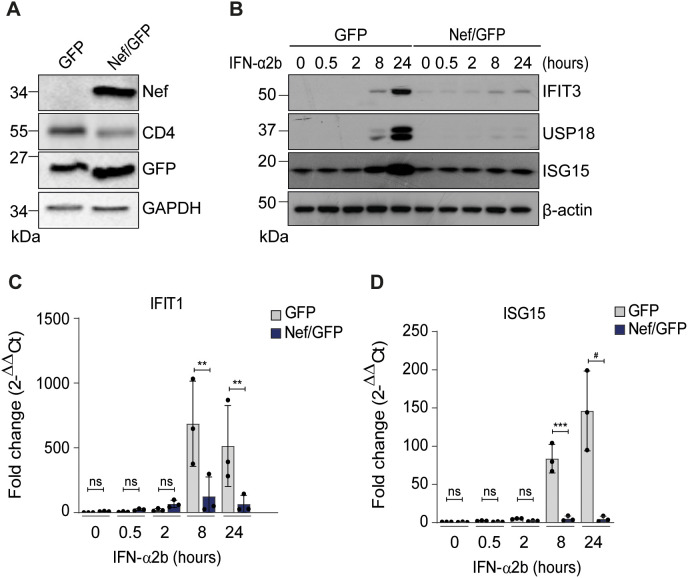
The HIV-1 Nef protein inhibits ISG expression. **A)** A3.01 T lymphocytes CD4^+^ expressing GFP alone (GFP) or Nef and GFP (NefWT/GFP) were obtained by lentiviral transduction using the MSCV retroviral vector containing a bicistronic IRES. Transduced cells were cultured for 72 h and sorted for GFP-positive by cell sorting. Next, the cells were lysed and analyzed by SDS-PAGE and western blot. **(B)** A3.01 T lymphocytes GFP or NefWT/GFP were treated with 1,000 IU/mL IFNα-2b for 0.5 h, 2 h, 8 h, or 24 h, or left untreated as a control (0 h). Next, cells were lysed, and their protein content was analyzed by western blot. **C-D)** A3.01 T lymphocytes GFP or NefWT/GFP were incubated with 1,000 IU/mL IFN-ɑ2b for 0.5 h, 2 h, 8 h, or 24 h, or left untreated as a control (0 h). After treatment, relative amounts of *IFIT1* **(C)** and *ISG15* **(D)** mRNA were analyzed by RT-qPCR. The absence of IFN-ɑ2b (0 h) was used to normalize mRNA levels. Results are means ± SD of data from three independent experiments. ** represents p < 0.01, *** = p < 0.001 and # = p < 0.0001. Statistical analysis was performed by two-way ANOVA followed by Bonferroni’s post hoc test. Representative images from three independent experiments.

ISG products may act as virus restriction factors that limit HIV-1 dissemination [43]. HIV-1 must counteract these host restriction factors to evade the innate immune response and establish a productive infection. Thus, we sought to investigate whether Nef affects JAK/STAT-triggered ISG expression. To this end, A3.01 CD4^+^ T cells were transduced with IRES-based retroviral vectors to express HIV-1 NL4−3 Nef and GFP (Nef/GFP) or a control to express GFP alone (GFP) and selected by cell sorting [42]. The cell viability was analyzed in both cases (S1A and S1B Figs), and Nef’s functionality was confirmed by the induced decrease of CD4 total levels in Nef/GFP cells (Fig 1A). As expected, IFN-α2b treatment of control T cells induced the expression of ISG such as IFIT3, USP18, and ISG15 in a time-dependent manner (Fig 1B). However, the presence of Nef prevented the induction of ISG expression under IFN-α2b stimulation (Fig 1B). Similar inhibitory effects were observed for IFIT1 and ISG15 induced expression at the mRNA level (Figs 1C and 1D). These results indicate that Nef hinders the transcription of ISGs and thus prevents the full activation of the JAK/STAT signaling pathway after IFN-α stimulus.

### Nef expression prevents the increase of pSTAT1 levels upon IFN-α stimuli

**Fig 2:**
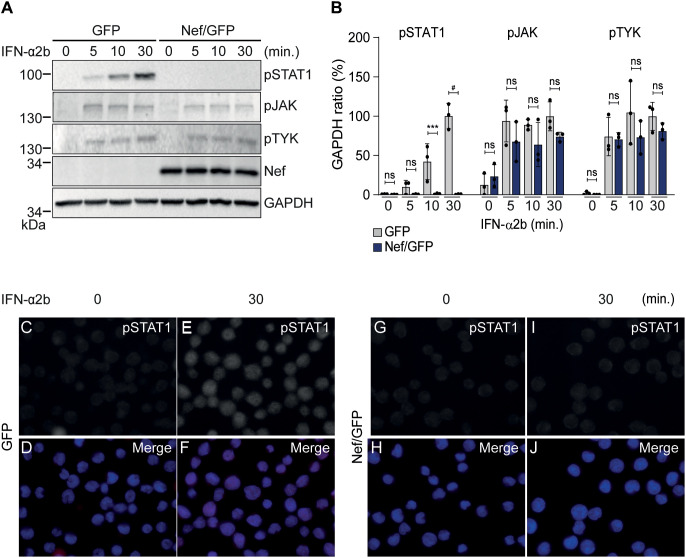
Nef reduces pSTAT1 levels in IFN-I-stimulated T cells. **A)** A3.01 T lymphocytes GFP or NefWT/GFP were incubated with 1,000 IU/mL IFN-ɑ2b for 5 min., 10 min., 30 min. or left untreated as a control (0 h). Next, the cells were lysed and their protein content was analyzed by SDS-PAGE and western blot. **B)** Densitometric values are expressed as pSTAT1, pJAK, and pJAK/GAPDH ratio. Results are means ± SD of data from three independent experiments. *** represents p < 0.001 and # = p < 0.0001. Statistical analysis was performed by two-way ANOVA followed by Bonferroni’s post hoc test. **C-J)** A3.01 T lymphocytes GFP or NefWT/GFP were incubated with 1,000 IU/mL IFN-ɑ2b for 30 min. or left untreated as a control (0 h). After treatment, cells adhered to the coverslips were fixed with ice-cold 100% methanol for 3 minutes and immunostained with Anti-STAT1 (phosphoY701) antibody, followed by immunostaining with secondary antibody conjugated with Alexa-594. Nuclei were stained with DAPI (blue). Coverslips were analyzed by fluorescence microscopy. Representative images from two independent experiments.

The nuclear translocation of the ISGF3 complex, composed of phosphorylated STAT1, STAT2, and IRF9, is crucial for IFN-α-induced expression of ISGs [44,45]. Therefore, we sought to confirm previous reports indicating that Nef inhibits STAT1 phosphorylation [20]. In control (GFP) cells, 5−30 minutes treatment with IFN-α2b induced STAT1 phosphorylation at Tyr701 (pSTAT1) (Figs 2A and 2B), accompanied by its nuclear translocation (Figs 2C-F). In contrast, STAT1 phosphorylation (Figs 2A and 2B) and nuclear accumulation of pSTAT1 (Figs 2G-J) were undetectable in A3.01 T cells expressing HIV-1 NL4−3 Nef. Similarly, nuclear accumulation of pSTAT1 following 30 min of IFN-α2b stimulation was also strongly inhibited in A3.01 T cells expressing SIVmac239 Nef (S2A-D Fig).

To gain insight into the mechanism by which Nef affects the JAK/STAT signaling pathway, we examined other signaling steps upstream of STAT1 phosphorylation. Essential steps of this pathway are the phosphorylation of the receptor kinases (JAK1 and TYK2), which phosphorylate the transcription factors STAT1 and STAT2. Western blot results showed that Nef expression did not prevent JAK1 or TYK2 phosphorylation after 5–30 minutes of stimulus (Figs 2A and 2B).

### Nef depletes the total levels of STAT1 in A3.01 T cells

**Fig 3:**
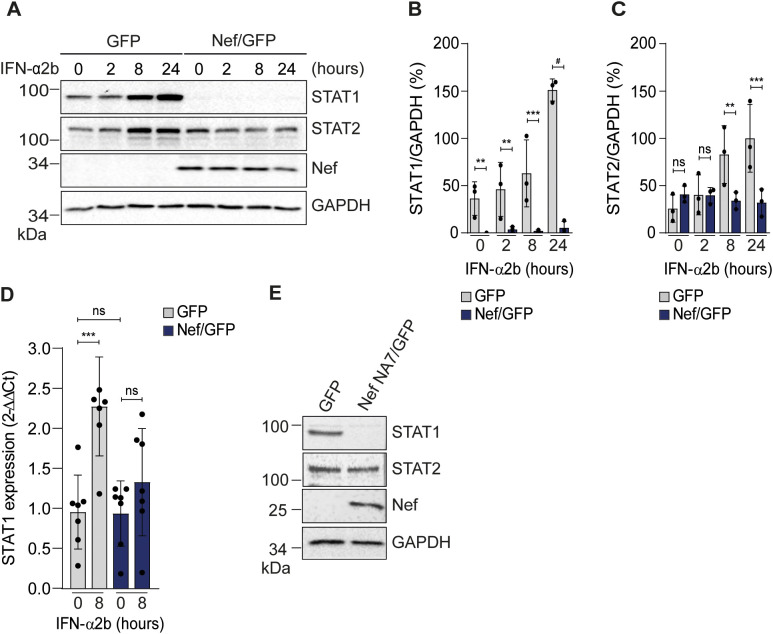
Nef depletes total STAT1 levels in T cells. **A)** A3.01 T lymphocytes CD4^+^ GFP or NefWT/GFP were incubated with 1,000 IU/mL IFNα-2b for 2 h, 8 h, 24 h or left untreated as a control (0 h). Next, the cells were lysed and their protein content was analyzed by SDS-PAGE and western blot. Densitometric values are expressed as **B)** STAT1/GAPDH ratio and **C)** STAT2/GAPDH ratio. **D)** A3.01 T lymphocytes GFP or NefWT/GFP were incubated with 1,000 IU/mL IFN-ɑ2b for 8 h or left untreated as a control (0 h). After treatment, relative amounts of *STAT1* mRNA were analyzed by RT-qPCR. The absence of IFN (0 h) was used to normalize mRNA levels. Results are means ± SD of data from three independent experiments. ** represents p < 0.01 and *** = p < 0.001. Statistical analysis was performed by two-way ANOVA followed by Bonferroni’s post hoc test. **E)** A3.01 T lymphocytes GFP or NefNA7/GFP were lysed and analyzed by SDS-PAGE and western blot. Representative results from three independent experiments.

Since pSTAT1 was not detectable in Nef positive cells upon IFN-α stimulation, we asked whether Nef alters the total levels of STAT1 and STAT2. Non-stimulated control (GFP) cells show detectable basal levels of total STAT1 and STAT2, that increased after 8 h or 24 h of IFN-α-stimulation, as expected for ISGs (Figs 3A-3C). Strikingly, Nef caused a strong reduction in the basal levels of STAT1, which did not increase even after 8 h or 24 h of IFN-α-stimulation (Figs 3A and 3B). Although, the basal levels of STAT2 were not affected by Nef, it also did not increase in response to longer periods of IFN-α-stimulation, confirming that ISG induction is impaired in Nef cells (Figs 3A and 3C).

To determine if Nef affects STAT1 expression at the transcriptional level, we performed qRT-PCR to compare the basal levels of STAT1 mRNA in GFP and NefWT/GFP cells. The results showed that Nef expression does not affect the constitutive levels of STAT1 mRNA (Fig 3D). However, Nef did inhibit the increase in STAT1 mRNA levels upon 8 h of IFN-α-stimulation (Fig 3D), confirming the Nef-mediated inhibition of the signaling pathway. Together, the results indicate the Nef reduces the total levels of STAT1 via a post-transcriptional mechanism.

Next, we asked if the capacity to deplete STAT1 is conserved for Nef NA7, encoded by a natural HIV-1 *nef* allele [41]. To this end, a pool of A3.01 CD4^+^ T cells expressing NefNA7 and GFP (NefNA7/GFP) was obtained (S1B Fig), and the total levels of STAT1 and STAT2 was analyzed by western blot. Similar to Nef NL4−3, the Nef NA7 allele induced a strong decrease in the total levels of STAT1 without changing the total levels of STAT2 (Fig 3E). Importantly, total STAT1 levels were also markedly reduced in A3.01 T cells expressing SIVmac239 Nef (S2D Fig), indicating that STAT1 depletion is not restricted to HIV-1 Nef and is conserved in Nef proteins from other primate lentiviruses. Together, these findings suggest that Nef-mediated antagonism of JAK/STAT signaling through STAT1 depletion is conserved among primate lentiviruses.

**Fig 4:**
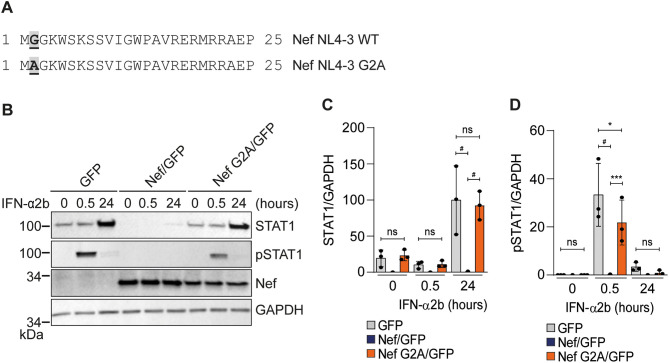
Association of Nef to cell membranes is required for STAT1 depletion in T cells. **A)** The amino acid sequence of the N-terminal part of Nef shows that a guanine at position 2, highlighted in bold, has been replaced individually by an alanine. The corresponding mutant is named NefG2A. **B)** A3.01 T lymphocytes CD4^+^ expressing GFP, Nef/GFP or NefG2A/GFP were stimulated with 1,000 IU/mL of IFN-ɑ2b for 30 min., 24 h, or left untreated as a control (0 h). Then, the cells were lysed and analyzed by SDS-PAGE and western blot. Densitometric values are expressed as **C)** STAT1/GAPDH ratio and **D)** pSTAT1/GAPDH ratio. Results are means ± SD of data from three independent experiments. * represents p < 0.05, *** = p < 0.001 and # = p < 0.0001. Statistical analysis was performed by two-way ANOVA followed by Bonferroni’s post hoc test.

STAT1 and STAT2 were shown to be associated to the cytoplasmic tail of IFNAR2 at the plasma membrane [46], and most Nef activities rely on its association to membranes, which is mediated by N-terminal myristoylation [47]. To test if the myristoylation domain in Nef is important in reducing STAT1 levels, we worked with a pool of A3.01 CD4^+^ T cells expressing Nef carrying a single mutation (a substitution of a glycine amino acid residue by an alanine residue at position 2) in the myristoylation site and GFP (NefG2A/GFP) (Fig 4A and S1B Fig) and checked the total STAT1 and pSTAT1 levels upon of IFNα stimulation. We found that a myristylation site is needed for efficient Nef-mediated downregulation of STAT1 (Fig 4B-4D).

### Nef induces proteasomal degradation of STAT1

**Fig 5:**
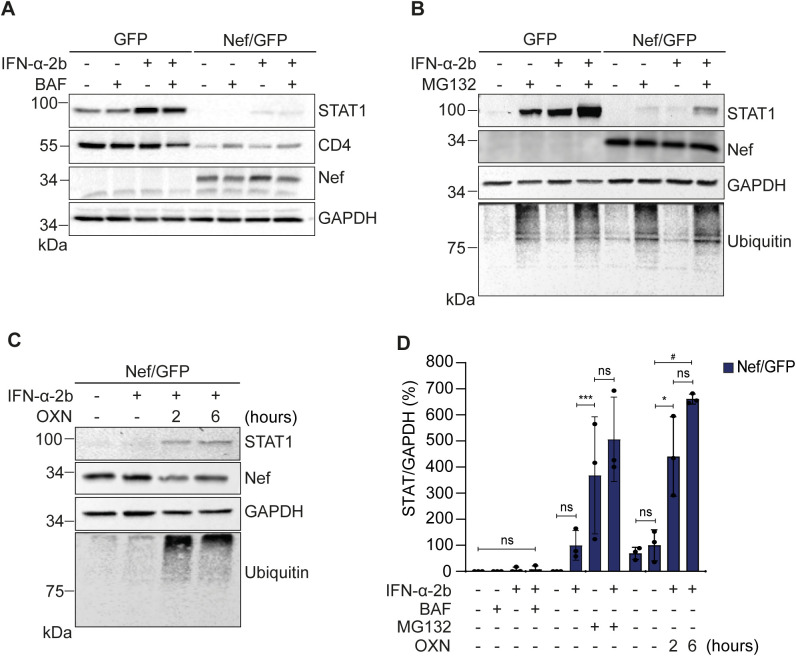
Nef induces STAT1 proteasomal degradation. **A)** A3.01 T lymphocytes CD4^+^ GFP or NefWT/GFP were incubated with 1,000 IU/mL IFN-ɑ2b for 24 h or left untreated as a control (0 h). After the first 18 h of treatment with IFN-ɑ2b, the cell culture medium was replaced with fresh medium supplemented with BAF (100 μM/mL) and 1,000 IU/mL IFN-ɑ2b. Cells were maintained in this supplemented medium for 6 h. Then, the cells were lysed and analyzed by SDS-PAGE and western blot. **B)** A3.01 T lymphocytes CD4 GFP or NefWT/GFP were incubated with 1,000 IU/mL IFN-ɑ2b for 24 h or left untreated as a control (0 h). After the first 18 h of treatment with IFN-ɑ2b, the cell culture medium was replaced by a new medium supplemented with MG132 (20 μM/mL) and 1,000 IU/mL IFN-ɑ2b. These cells were maintained in this supplemented medium for 6 h, changing the medium after 3 h. Then, the cells were lysed and analyzed by SDS-PAGE and western blot. **C)** A3.01 T lymphocytes CD4^+^ GFP or NefWT/GFP were incubated with 1,000 IU/mL IFN-ɑ2b for 24 h or left untreated as a control (0 h). After the first 18 or 22 h of treatment with IFN-ɑ2b, the cell culture medium was replaced by a new medium supplemented with OXN (200 nM/mL) and 1,000 IU/mL IFN-ɑ2b. These cells were maintained in this supplemented medium for 6 or 2 h. Then, the cells were lysed and analyzed by SDS-PAGE and western blot. **D)** Densitometric values are expressed as STAT1/GAPDH ratio. Results are means ± SD of data from three independent experiments. * represents p < 0.05, *** = p < 0.001 and # = p < 0.0001. Statistical analysis was performed by two-way ANOVA followed by Bonferroni’s post hoc test.

As the depletion of STAT1 by Nef is likely post-translational (Fig 3D), we sought to determine which cellular protein degradation pathway is employed by Nef to reduce STAT1 levels. To this end, we treated CD4^+^ T lymphocytes with either bafilomycin A1 (BAF), a lysosomal acidification inhibitor, MG132, a broad proteasome inhibitor, or ONX-0914 (OXN), an immunoproteasome-specific inhibitor [48] as indicated in S3A-D Fig. Treatment with BAF did not restore STAT1 levels in Nef cells (Figs 5A and 5D), but alleviated Nef-mediated CD4 degradation, known to be lysosomal dependent [22]. In contrast, treating CD4^+^ T lymphocytes with MG132 (Figs 5B and 5D) or ONX (Figs 5C and 5D), which led to the accumulation of ubiquitinated proteins due to impaired proteasomal degradation, partially restored the levels of STAT1 in Nef cells under IFN-α. These results strongly indicate that STAT1 depletion by Nef involves the proteasomal pathway rather than lysosomes.

## Discussion

The JAK/STAT signaling pathway is crucial in regulating viral infections, and different virus families developed strategies to hinder its activation (Reviewed by [49]). These strategies include inhibition of phosphorylation [50,51], alterations in subcellular localization [52], induced-degradation of signaling proteins [53–55], or prevents the association of STAT1 with an activated IFN receptor [56]. Our study contributes to this knowledge by showing that the HIV-1 Nef protein downmodulates the activation of the JAK/STAT signaling pathway by inducing STAT1 proteasomal degradation in CD4^+^ T lymphocytes.

The JAK/STAT signaling pathway is known to interfere with HIV-1 infection, and Nef and other HIV-1 accessory proteins directly counteract ISG products, such as IFITM [42,57], SERINC 3/5 [58,59] and BST-2 [60,61], through post-translational mechanisms. Here, we provide evidence that Nef may play a broader inhibitory effect on IFN-α mediated antiviral response (Fig 1) by interfering with the JAK/STAT signaling cascade in T cells. This effect is not due to impaired activation of IFNAR1/2 associated kinases JAK1 or TYK2 since the levels of their phosphorylated forms upon IFN-α stimulation in Nef cells are similar to control cells (Fig 2). In contrast, pSTAT1 was undetectable in IFN-α stimulated cells expressing either HIV-1 or SIVmac 239 Nef (Fig 2 and S2 Fig), indicating that Nef targets the pathway downstream of receptor-associated kinase activation. Although HIV-1 Nef was previously shown to reduce pSTAT1 levels in CD4^+^ T lymphocytes [20], the mechanism responsible for this effect remained unknown.

Our data indicate that the absence of pSTAT1 in HIV-1 and SIVmac239 Nef-expressing cells following IFN-α stimulation is largely explained by the marked depletion of total STAT1 levels (Fig 3 and S2 Fig). Moreover, this reduction occurs via a post-transcriptional mechanism since STAT1 protein levels are reduced without a significant alteration in its total mRNA levels (Fig 3). Interestingly, HIV-1 Nef does not affect the basal levels of STAT2, a STAT1 interaction partner in the JAK/STAT signaling pathway, suggesting a specific activity of Nef on STAT1 (Fig 3). The absence of STAT1 in these cells could potentially impact other biological processes, such as apoptosis, cell growth, and differentiation (reviewed in [62]), which may lead to cellular imbalances beyond antiviral defenses. Moreover, given the importance of STAT1 for the regulation of Th1/Th2 cell differentiation, via transcription factor T-bet, the degradation of STAT1 by Nef may interfere with the cell polarization process (reviewed in [63]).

Nef induces changes in subcellular localization and lysosomal degradation of several host cell transmembrane proteins (reviewed in [22]). However, we showed that, unlike what happens to CD4 or MHC-I [64,65], Nef-induced depletion of STAT1 does not depend on lysosomal activity (Fig 5). In contrast, inhibition of proteasomal activity alleviated STAT1 downregulation by Nef. The utilization of the proteasomal machinery by Nef is uncommon; however, it has been previously documented in the context of Nef-mediated p53 degradation, which occurs through its interaction with the ubiquitin E3 ligase E6AP [66,67]. In macrophages treated with IFN-γ, STAT1 interacts with ubiquitin E3 ligase enzyme SMURF1 (Smad E3 ligase of the HECT type 1) [68], leading to ubiquitination and subsequent proteasomal degradation. Since SMURF1 is expressed in CD4^+^ T lymphocytes [69], it represents an interesting target for further investigation.

In this study, we employed the strategy of inhibiting proteasomal activity using two different compounds: MG132, a broad proteasome inhibitor, and ONX, an inhibitor specifically targeting the catalytic subunit β5i (LPM7) of the immunoproteasome [48,70,71]. Both constitutive proteasome (CP) and immunoproteasome (IP) play essential roles in maintaining cellular proteostasis, differing in their structure and expression patterns. The CP, expressed in all cells, comprises catalytic subunits β1 (LPM2), β2 (MECL-1), and β5 (LPM7). In contrast, the IP, expressed in cells of the hematopoietic lineage, presents homologous subunits known as β1i (LPM2), β2i (MECL-1), and β5i (LPM7) [72,73]. While the precise functional distinctions between CP and IP are not fully understood, the IP is recognized for its importance in MHC-I assembly and peptide presentation for cytotoxic T lymphocytes [74].

The Nef protein undergoes N-terminal myristoylation, facilitating its association with the cytosolic side of cellular membranes [47]. This modification is a prerequisite for many of Nef’s described cellular functions. The depletion of STAT1 by Nef, described in this study, seems to require its membrane association since the integrity of Nef’s myristylation domain was necessary for efficient STAT1 depletion (Fig 4). This hypothesis fits with the structural model recently proposed for the IFNAR2/JAK1/STAT signaling complex, in which STAT1 and STAT2 would be associated with the cytoplasmic tail of IFNAR2, on the inner side of the plasma membrane, prior to stimulation with IFN-I [46]. This does not rule out the possibility of Nef’s activity on STAT1, which may also occur in endosomal membranes following endocytosis of the signaling complex [75,76]. Similarly, myristoylation of Nef was found to be essential for modulating the activation of Tec family kinases Itk and Btk, which play a critical role in antigen receptor signaling in T and B cells. When Nef was mutated in the myristoylation domain (NefG2A), it was unable to localize to the plasma membrane and failed to induce the same activation levels of Itk and Btk kinases as observed with wild-type Nef (NefWT). Notably, NefG2A retained the ability to form a complex with both kinases in the cytoplasm, highlighting the importance of myristoylation for proper membrane recruitment and kinase activation [36].

The data presented in this study reveal that Nef reduces STAT1 levels through post-translational mechanisms, specifically by inducing its proteasomal degradation. This impedes the full activation of the JAK/STAT signaling pathway by IFN-I, consequently limiting the expression of antiviral ISGs in A3.01 T cells. Further investigation is necessary to elucidate the cellular factors hijacked by Nef to mediate STAT1 downregulation.

## Supporting information

S1 Fig Cell viability and GFP expression analysis. A3.01 CD4^+^ T cells expressing GFP, NefWT/GFP, NefNA7/GFP or NefG2A/GFP and screened for cell viability analysis, using the FVS575V reagent, and for GFP expression, by flow cytometry. **A)** Dot plots showing population viability of live cells cultured for 24 h at 37 °C in the presence or absence of 1,000 IU/mL IFN-α2b. **B)** Histogram showing GFP fluorescence intensity in GFP, NefWT/GFP, NefNA7/GFP, and NefG2A/GFP cells analyzed by flow cytometry.
(TIF)

S2 Fig SIVmac293 Nef reduces STAT1 levels in IFN-I-stimulated T cells. **A-C)** A3.01 T lymphocytes expressing GFP, NefWT/GFP or NefSIVmac293/GFP were incubated with 1,000 IU/mL IFN-ɑ2b for 30 min. or left untreated (0 h).Cells were then fixed with ice-cold 100% methanol for 3 min and immunostained with an anti-phospho-STAT1 (Try701) antibody, followed by an Alexa-594 conjugated secondary antibody. Nuclei were stained with DAPI (blue). Coverslips were analyzed by fluorescence microscopy. **D)** A3.01 T lymphocytes expressing GFP, NefWT/GFP or NefSIVmac293/GFP, using ires-based constructs, were incubated with 1,000 IU/mL IFNα-2b for 24 h or left untreated (0 h). Cells were then lysed and protein extracts were analyzed by SDS-PAGE and western blot to determine total STAT1 levels.
(TIF)

S3 Fig Drugs treatment timeline. Schematic for timeline cell treatment with **A)** BAF (100μM/mL) and 1,000 IU/mL IFN-ɑ2b, **B)** MG132 (20 μM/mL) and 1,000 IU/mL IFN-ɑ2b, **C)** OXN (200 Nm/mL) and 1,000 IU/mL IFN-ɑ2b for 2 h, **D)** OXN (200 Nm/mL) and 1,000 IU/mL IFN-ɑ2b for 6 h.
(TIF)

S1 Raw Images Unprocessed SDS-PAGE and western blot images. (PDF)

S1 Data Excel Spread Sheet containing the numeric data for Figs 1C, 1D, 2B, 3B, 3C, 3D, 4C, 4D and 5D. (XLSX)
