# Supplementary figures and images for: HIV-1 Nef inhibits the JAK/STAT signaling pathway by inducing proteasomal degradation of STAT1
Source: PLoS One. 2026 Jul 10;21(7):e0352649. doi: 10.1371/journal.pone.0352649 (PMC13353947; doi:10.1371/journal.pone.0352649)

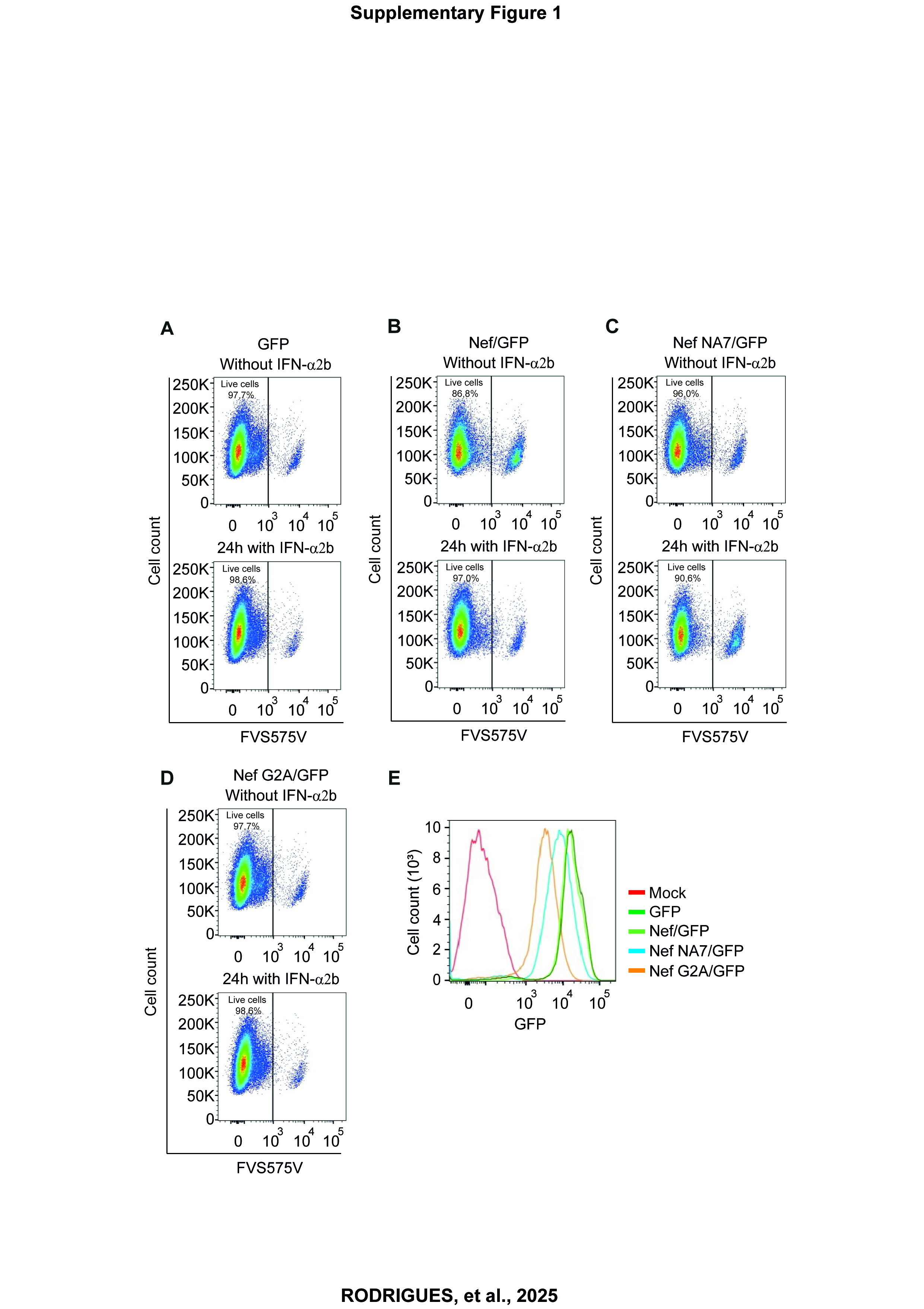

Supplement: S1 Fig — A3.01 CD4+ T cells expressing GFP, NefWT/GFP, NefNA7/GFP or NefG2A/GFP and screened for cell viability analysis, using the FVS575V reagent, and for GFP expression, by flow cytometry. A) Dot plots showing population viability of live cells cultured for 24 h at 37 °C in the presence or absence of 1,000 IU/mL IFN-α2b. B) Histogram showing GFP fluorescence intensity in GFP, NefWT/GFP, NefNA7/GFP, and NefG2A/GFP cells analyzed by flow cytometry. (TIF) [file pone.0352649.s001.tif]

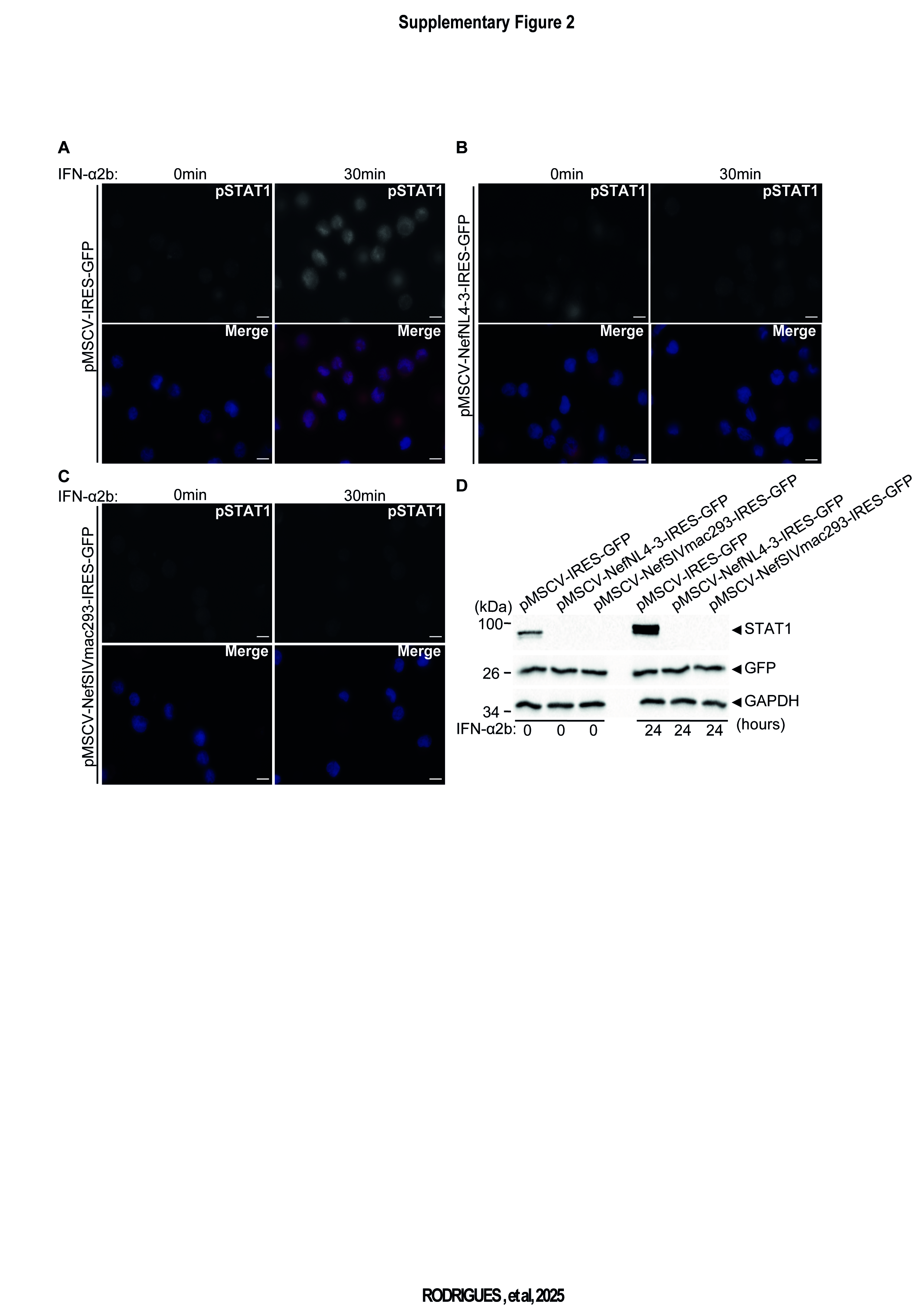

Supplement: S2 Fig — A-C) A3.01 T lymphocytes expressing GFP, NefWT/GFP or NefSIVmac293/GFP were incubated with 1,000 IU/mL IFN-ɑ2b for 30 min. or left untreated (0 h).Cells were then fixed with ice-cold 100% methanol for 3 min and immunostained with an anti-phospho-STAT1 (Try701) antibody, followed by an Alexa-594 conjugated secondary antibody. Nuclei were stained with DAPI (blue). Coverslips were analyzed by fluorescence microscopy. D) A3.01 T lymphocytes expressing GFP, NefWT/GFP or NefSIVmac293/GFP, using ires-based constructs, were incubated with 1,000 IU/mL IFNα-2b for 24 h or left untreated (0 h). Cells were then lysed and protein extracts were analyzed by SDS-PAGE and western blot to determine total STAT1 levels. (TIF) [file pone.0352649.s002.tif]

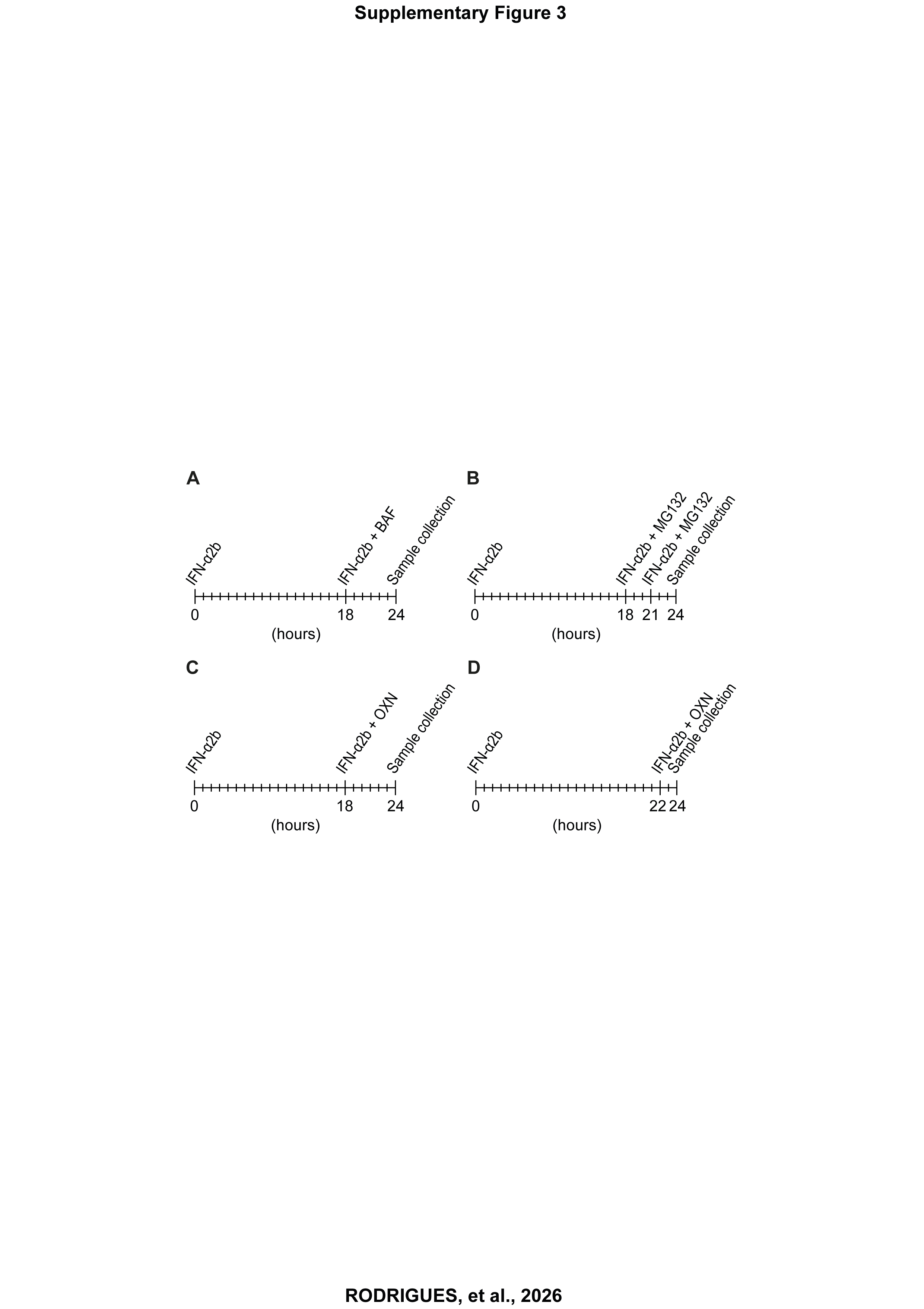

Supplement: S3 Fig — Schematic for timeline cell treatment with A) BAF (100μM/mL) and 1,000 IU/mL IFN-ɑ2b, B) MG132 (20 μM/mL) and 1,000 IU/mL IFN-ɑ2b, C) OXN (200 Nm/mL) and 1,000 IU/mL IFN-ɑ2b for 2 h, D) OXN (200 Nm/mL) and 1,000 IU/mL IFN-ɑ2b for 6 h. (TIF) [file pone.0352649.s003.tif]
