# Supplementary material for: HIV-1 Nef inhibits the JAK/STAT signaling pathway by inducing proteasomal degradation of STAT1
Source: PLoS One. 2026 Jul 10;21(7):e0352649. doi: 10.1371/journal.pone.0352649 (PMC13353947; doi:10.1371/journal.pone.0352649)

S4. Raw Images. Unprocessed western blot and SDS-PAGE gel images

Unprocessed western blot images corresponding to figure 1A.

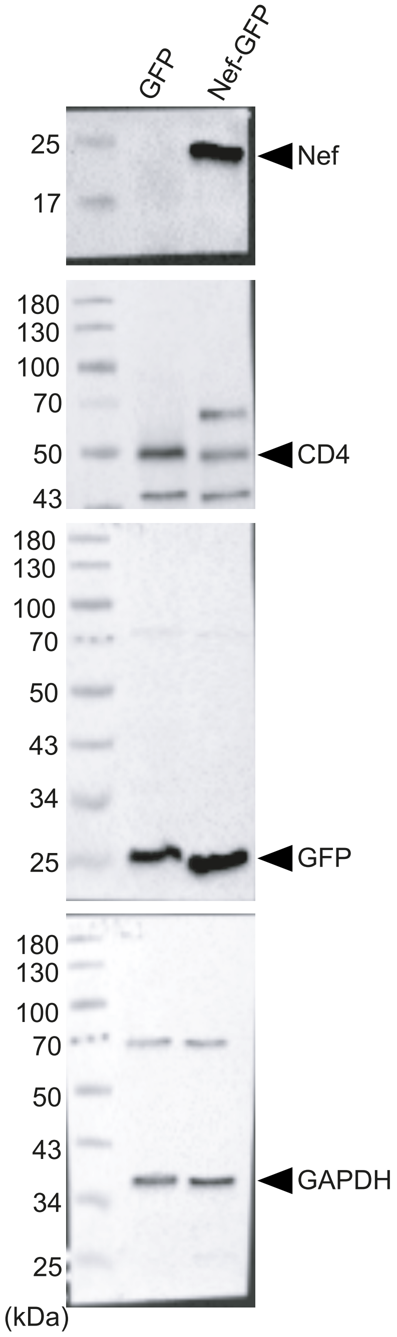

S4. Raw Images. Unprocessed western blot and SDS-PAGE gel images

Unprocessed western blot images corresponding to figure 2A.

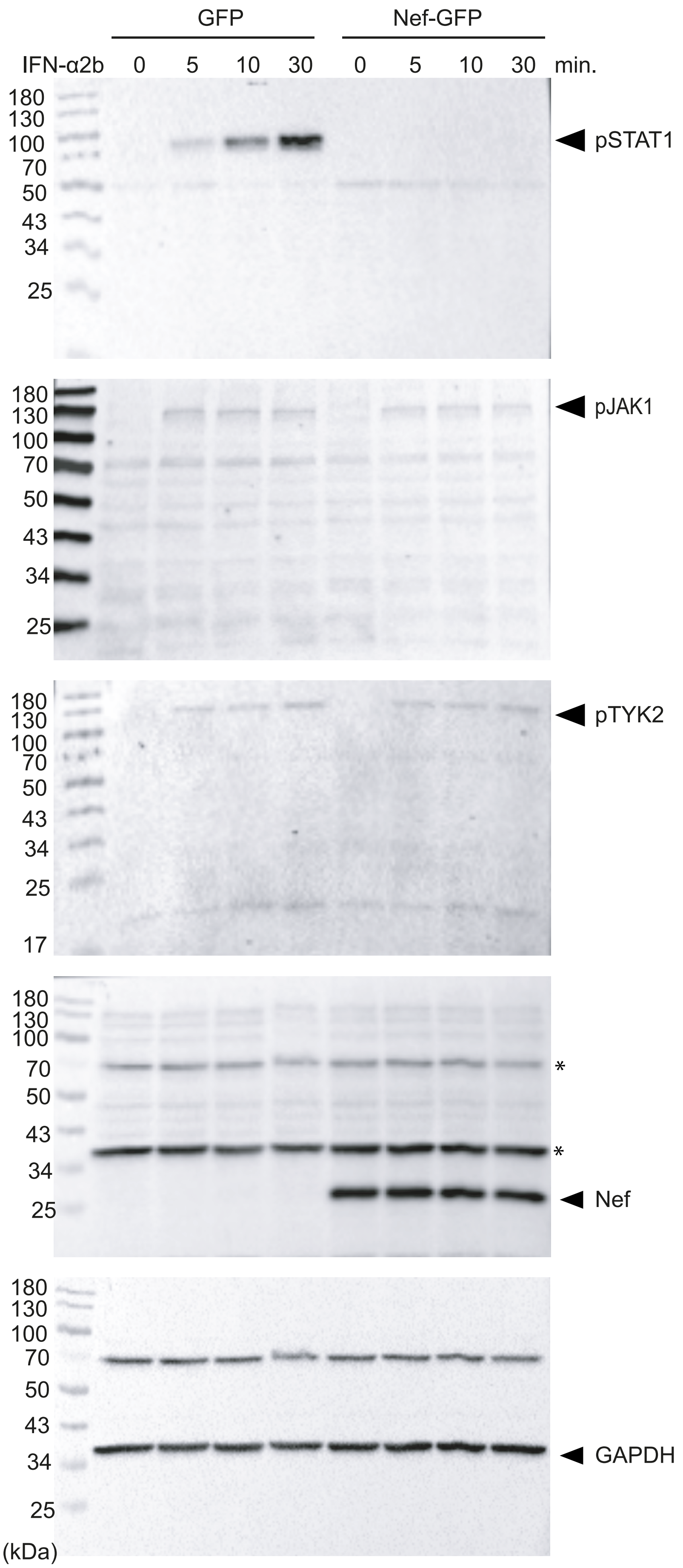

*The membrane was re-probed after using the anti-GAPDH antibody.

S4. Raw Images. Unprocessed western blot and SDS-PAGE gel images

Unprocessed western blot images corresponding to figure 3A.

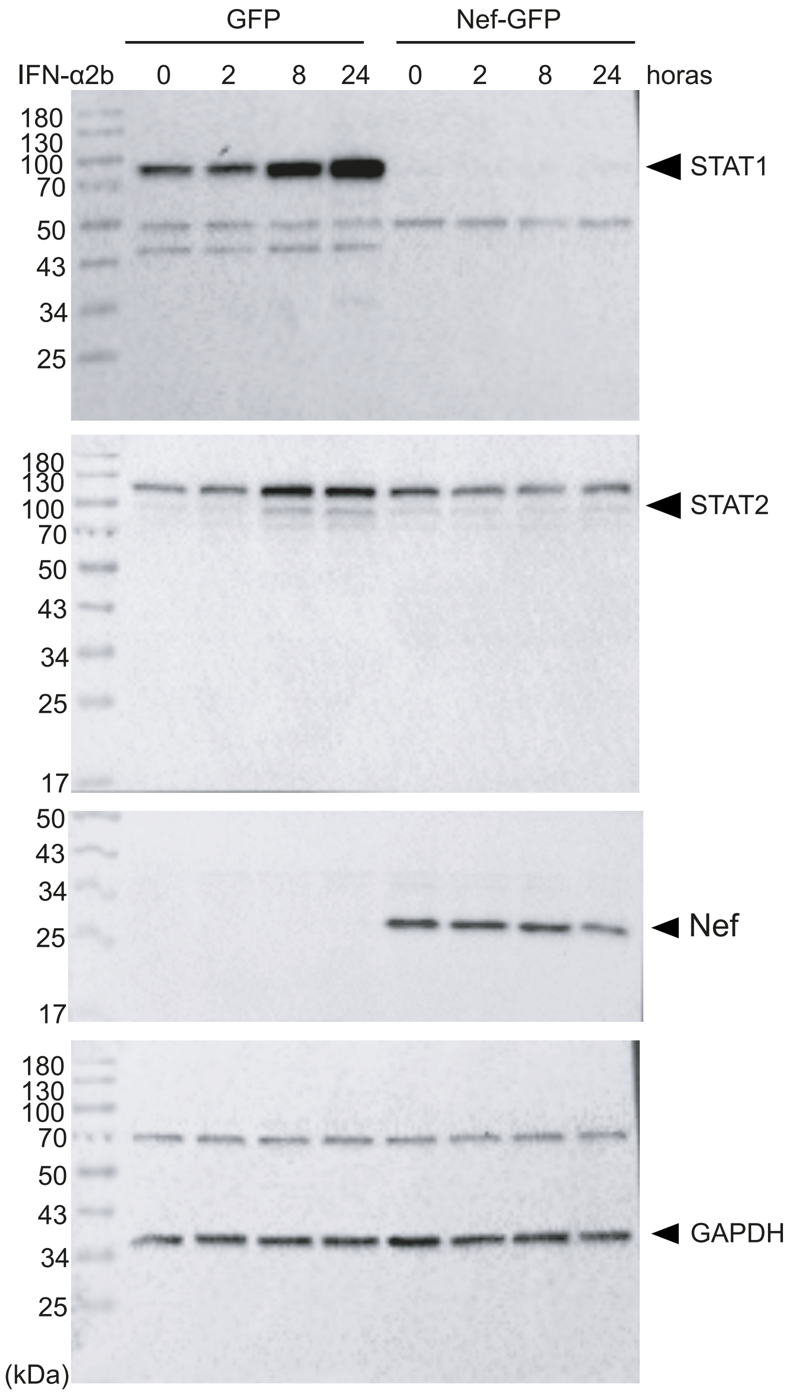

S4. Raw Images. Unprocessed western blot and SDS-PAGE gel images

Unprocessed western blot images corresponding to figure 3E.

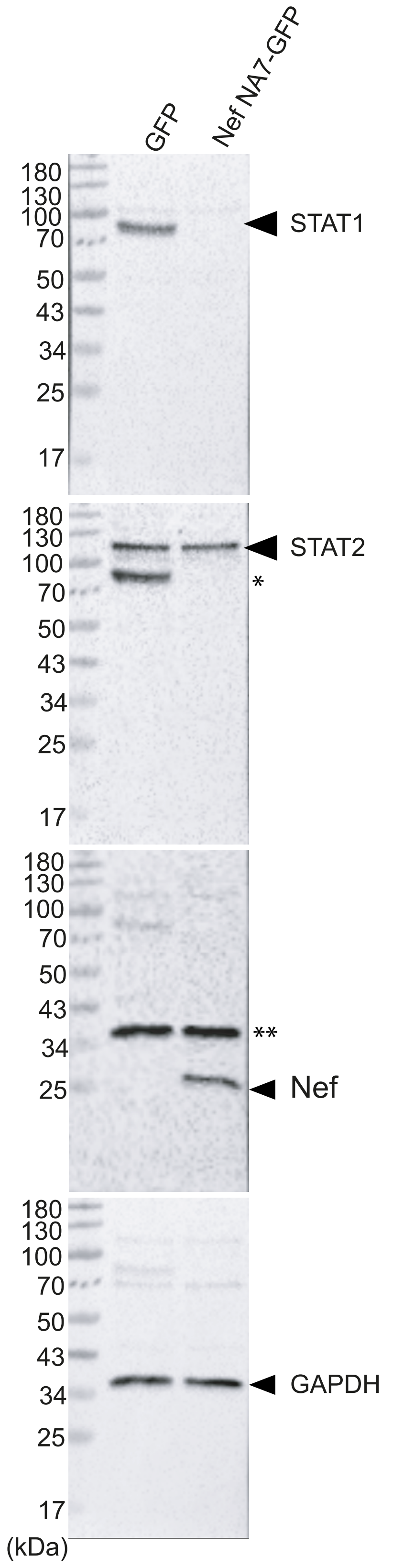

*The membrane was re-probed after using the anti-STAT1 antibody.

**The membrane was re-probed after using the anti-GAPDH antibody.

S4. Raw Images. Unprocessed western blot and SDS-PAGE gel images

Unprocessed western blot images corresponding to figure 4A.

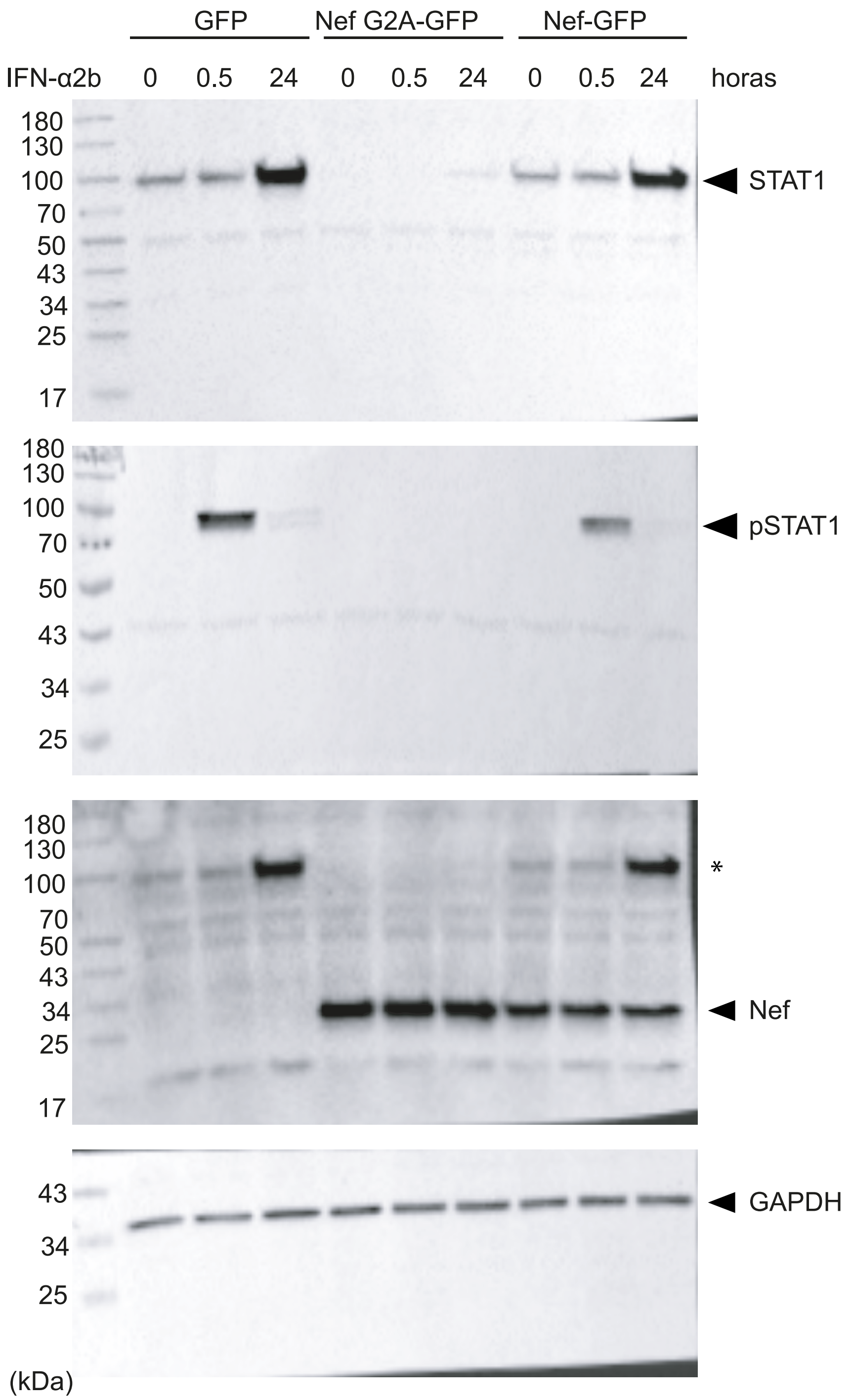

*The membrane was re-probed after using the anti-STAT1 antibody.

S4. Raw Images. Unprocessed western blot and SDS-PAGE gel images

Unprocessed western blot images corresponding to figure 5A.

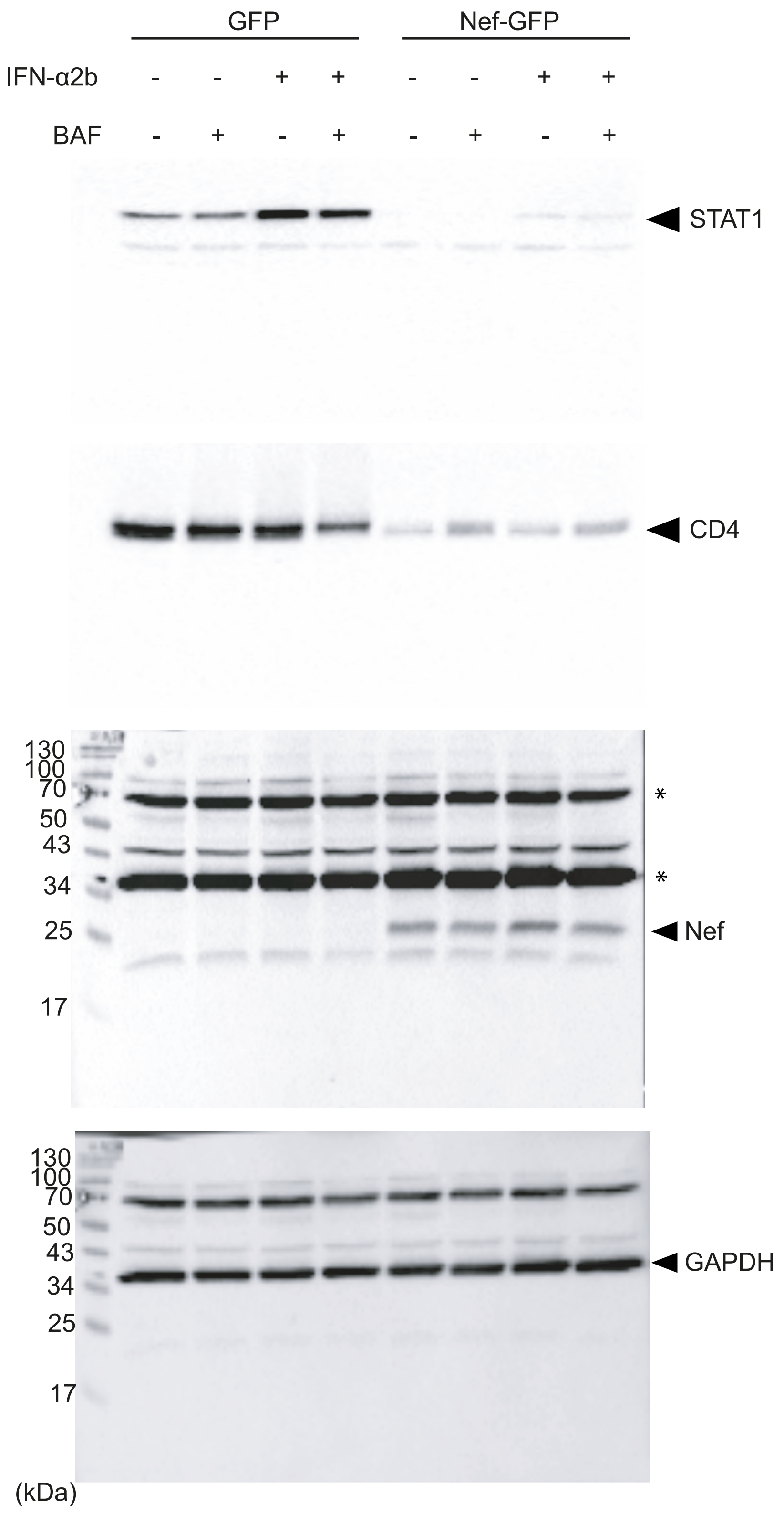

*The membrane was re-probed after using the anti-GAPDH antibody.

S4. Raw Images. Unprocessed western blot and SDS-PAGE gel images

Unprocessed western blot images corresponding to figure 5B.

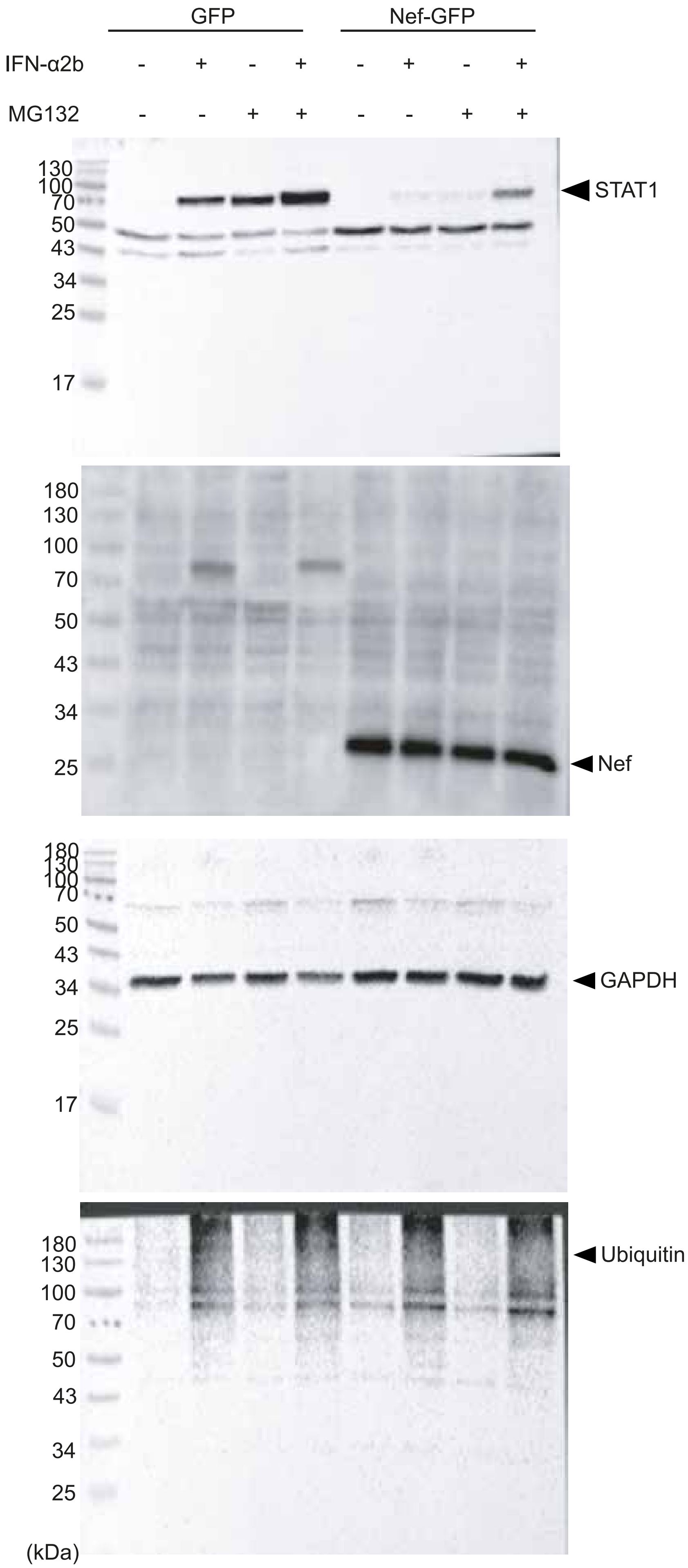

RODRIGUES, et al, 2026

S4. Raw Images. Unprocessed western blot and SDS-PAGE gel images

Unprocessed western blot images corresponding to figure 5C.

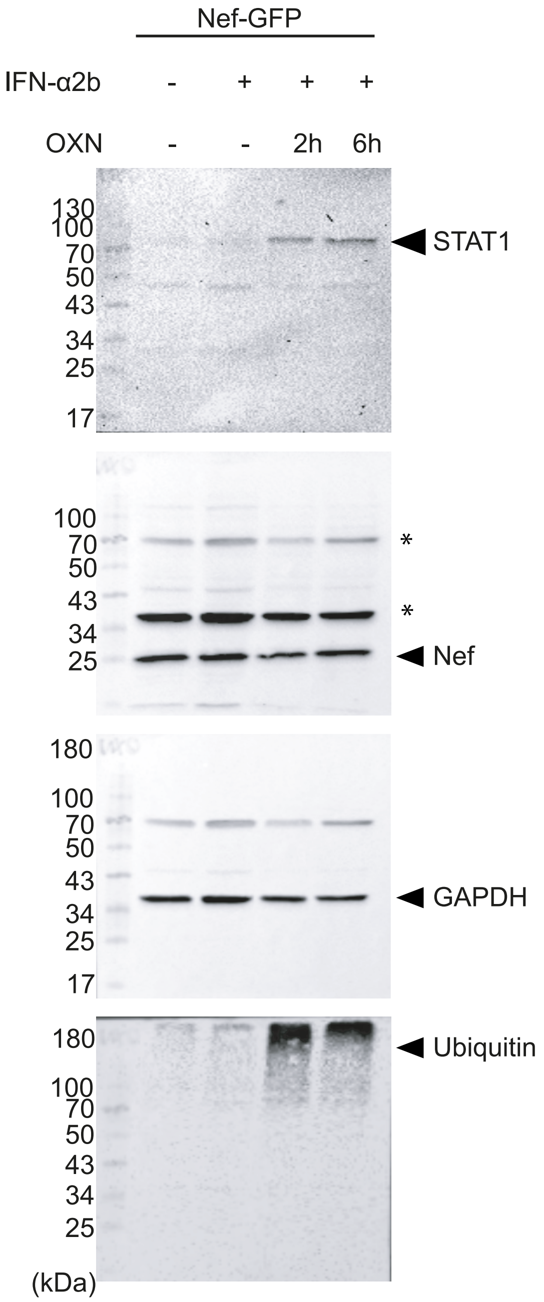

*The membrane was re-probed after using the anti-GAPDH antibody.

S4. Raw Images. Unprocessed western blot and SDS-PAGE gel images

Unprocessed western blot images corresponding to figure S2.

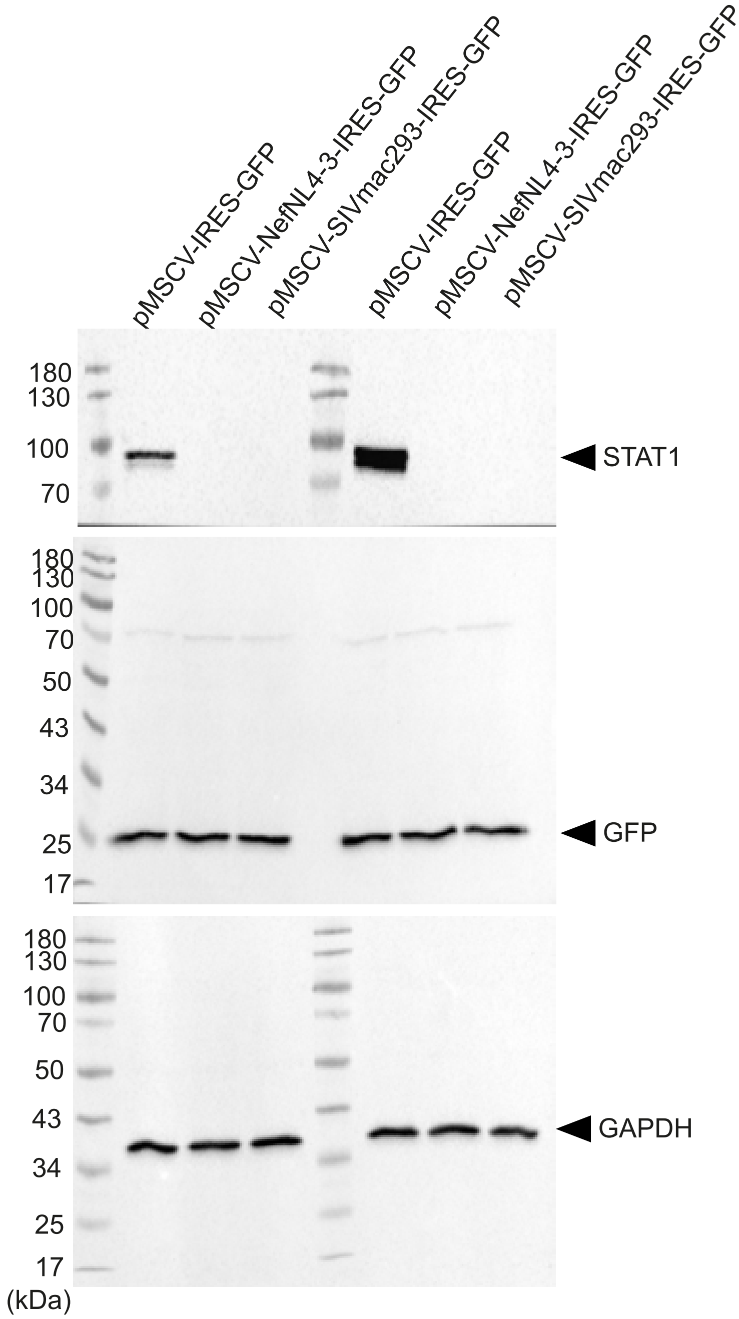

Supplement: S1 Raw Images — (PDF) [file pone.0352649.s004.pdf]
